# Bacterial cooperation in the wild and in the clinic: Are pathogen social behaviours relevant outside the laboratory?

**DOI:** 10.1002/bies.201200154

**Published:** 2012-12-27

**Authors:** Freya Harrison

**Affiliations:** School of Molecular Medical Sciences, University of NottinghamNottingham, United Kingdom

**Keywords:** cooperation, infectious disease, pathogens, social evolution, virulence

## Abstract

Individual bacterial cells can communicate via quorum sensing, cooperate to harvest nutrients from their environment, form multicellular biofilms, compete over resources and even kill one another. When the environment that bacteria inhabit is an animal host, these social behaviours mediate virulence. Over the last decade, much attention has focussed on the ecology, evolution and pathology of bacterial cooperation, and the possibility that it could be exploited or destabilised to treat infections. But how far can we really extrapolate from theoretical predictions and laboratory experiments to make inferences about ‘cooperative’ behaviours in hosts and reservoirs? To determine the likely importance and evolution of cooperation ‘in the wild’, several questions must be addressed. A recent paper that reports the dynamics of bacterial cooperation and virulence in a field experiment provides an excellent nucleus for bringing together key empirical and theoretical results which help us to frame – if not completely to answer – these questions.

## Introduction: Why study the social lives of bacteria?

Any behaviour that benefits another individual or individuals, and which is selected for at least in part because of this benefit, is classed as cooperation 1. In bacteria, many exoproducts can be seen as cooperative – perhaps the best known include molecules that harvest nutrients (such as elastase 2 and iron-scavenging siderophores 3), biofilm polymers 4, 5 and exotoxins (such as botulinum, anthrax and Shiga and cholera toxins 6). These all represent ‘public goods’ whose benefits can be enjoyed not just by the producing cells, but potentially by other cells in the vicinity regardless of their own level of production. As these molecules are metabolically costly to produce, populations of cooperating bacteria are open to exploitation by ‘cheating’ mutants, who enjoy the fruits of their neighbours' efforts but pay none of the associated costs 4, 7–11. This situation mirrors that in many animal species, where behaviours such as cooperative breeding, predator mobbing, and alarm calling confer shared benefits but entail temptations to defect 12.

How, when and why natural selection favours the evolution and maintenance of cooperation despite the advantages of cheating are therefore key questions in biology, and have received a great deal of theoretical and empirical attention (synthesised in 12–15). Bacteria are tractable model systems for testing ecological and evolutionary hypotheses about cooperation, but microbiologists are also interested in bacterial sociality in its own right because it often determines virulence (parasite-induced harm). For instance, siderophores are a necessary virulence factor in acute infections of the human opportunistic pathogen *Pseudomonas aeruginosa* 8, 16, biofilms confer enhanced resistance to antibiotics and host immune attack 17 and exotoxins are often necessary for successful colonisation of hosts – the Cry toxin of the insect pathogen *Bacillus thuringiensis*, for example, induces pore formation in gut epithelial cells, facilitating invasion of the host haemocoel (the body cavity, containing a mixture of blood, lymph and interstitial fluid) 18. Consequently, understanding the evolution of cooperation may improve our understanding of the evolution of pathogen virulence and suggest novel ways of treating bacterial infection 19, 20. For example, it has been suggested that cheating mutants could be used as ‘Trojan horses’ 21. By avoiding the costs of cooperation, cheating mutants can gain a fitness advantage over cooperators and increase in frequency until they dominate populations; inoculating infected hosts with engineered cheats could therefore lead to a predominance of cheats in the infection and reduced virulence; moreover, the cheats could be engineered to carry useful alleles (e.g. antibiotic susceptibility), which would hitch-hike to high frequency and so render the infection more susceptible to traditional prophylaxis. However, in order for this approach to work, we must be sure that the environment and population structure of the target pathogen is such that cheats can invade the population to a sufficiently high level to allow clearance upon administration of antibiotics.

## How cooperation evolves

What, then, do we know about the ecological conditions that favour cooperation versus cheating? Significant advances have been made in this field, mainly relying on concepts rooted in inclusive fitness theory 14, 22, 23. Inclusive fitness theory tells us that cooperation can be favoured in two non-mutually exclusive ways. First, over an individual's lifetime, cooperation can bring benefits that outweigh its short-term cost and cause a net increase in direct fitness (the individual leaves more offspring). For instance, cooperation may make an individual more likely to benefit from the cooperative acts of others 24, or may bring benefits via its ecological effects. One way this can happen in patch-structured populations (where the population is divided among discrete, localised patches of habitat) is if cooperation increases group size and the probability of an individual leaving descendants is a positive function of local group size, i.e. in a metapopulation where competition occurs mainly between patches 7, 22, 25. Second, if the beneficiaries of cooperation tend also to carry cooperative alleles, then they will leave more offspring; in this way, cooperative alleles increase in frequency regardless of the direct cost to the actor, whose indirect fitness is thus increased 7, 22. This can occur actively, through reciprocity or through preferential targeting of cooperation towards relatives 26 or individuals who carry distinctive phenotypic markers of cooperation (‘greenbeards’: 27–29), or passively through indiscriminate cooperation in conditions that tend to keep relatives together, such as limited dispersal between habitat patches, or dispersal in groups of relatives 25, 30–33. It is likely that the early emergence of cooperation relies on indirect fitness benefits resulting from high relatedness, or on direct benefits that accrue because the interests of the individual and group are aligned (e.g. if between-patch competition is more intense than within-patch competition). Later, the emergence of reciprocity, greenbeards, kin discrimination and policing mechanisms can act to maintain cooperation even if ecological or demographic change means that these early conditions are no longer met.

In order to determine whether bacterial behaviours shown to be cooperative in laboratory experiments are relevant to pathogen ecology, evolution and virulence in the wild, we must therefore ask three questions. First, is the behaviour really cooperative in hosts and/or reservoirs? Second, is natural population structure likely to favour cooperation or cheating? Finally, how do other selection pressures acting within hosts and/or reservoirs affect selection on cooperation?

## Are bacteria really cooperative in hosts and/or reservoirs?

Microbiologists know that it is not always easy to determine whether the phenotypes we see expressed by bacteria in the laboratory reflect those expressed inside a natural host. The chemical composition of the environment, temperature and growth mode (e.g. planktonic vs. surface-attached) all affect bacterial physiology and cue specific patterns of gene expression 34–37. When considering social traits, the situation becomes more difficult: not only must we ask whether this trait is expressed inside a host and whether it really aids persistence, we also have to ask whether the trait is actually still cooperative in this environment.

For instance, it is plausible that experiments in liquid growth medium allow cheats much greater access to diffusible public goods than they experience in nature. In chronic lung infections of people with cystic fibrosis, *P. aeruginosa* inhabits viscous, adhesive mucus, which likely retards the diffusion of secreted molecules. In the lab, increasing the viscosity of experimental growth medium and so retarding both cell migration and public goods diffusion prevents cheating mutants from overwhelming siderophore-producing populations of *P. aeruginosa* 30. It seems likely that this is partially due to siderophores being retained more closely in the vicinity of producing cells, i.e. siderophore production becomes less cooperative and more selfish as viscosity increases 30, 38. Such shifting of a trait's effect from the group to the individual will dramatically alter how natural selection acts upon it.

In a recent study, Raymond et al. 39 conducted a field experiment to determine whether *B. thuringiensis* Cry toxin truly represents a public good in semi-natural conditions. By inoculating a plot of cabbage plants with varying ratios of Cry^+^ and Cry^−^ spores, at a range of total densities, releasing host diamondback moth (*Plutella xylostella*) larvae onto the plants and recovering spores from cabbage leaves at intervals over a 56-day period, Raymond et al. were able to study the evolutionary dynamics of Cry production. Crucially, after initial inoculation, transmission of bacteria between hosts was allowed to occur naturally. This contrasts markedly with previous work on the evolution of cooperation, in which dispersal between environmental patches or transmission between hosts is controlled by the experimenter and experimental hosts are essentially treated as rather advanced test tubes. Raymond et al. found that the Cry toxin is just as social in the field as it is in the lab: in both settings, the proportion of Cry^−^ spores increases over the course of infection, demonstrating that non-producers can gain access to the haemocoel and grow inside the host at the expense of the producer cells. Further, as we would expect for a public good that enhances population growth 40, this advantage of cheating is negatively frequency-dependent: the more cheats there are present, the less Cry is available for cheats to exploit and the worse they do.

## Is natural population structure likely to favour cooperation or cheating?

The answer to this question depends on several variables, including but not limited to: multiplicity of infection (Are hosts infected by single clones or multiple genotypes?), resource supply within infected hosts (How necessary is cooperation for growth?), the relationship between virulence and transmission (How does growth rate or yield within a host affect transmission probability?), host population structure (How frequent are opportunities for transmission?) and transmission mode (direct or via an environmental reservoir, where population structure may differ from that in hosts). Taking these factors into account can produce very different predictions of the evolution of virulence than those resulting from simple laboratory experiments 41. Moreover, extensive theoretical work illustrates how both pathogen and host population structure mediate selection for virulence (e.g. 42); if virulence is determined by cooperation, these variables will by necessity also affect pathogen sociality.

**Figure 1:**
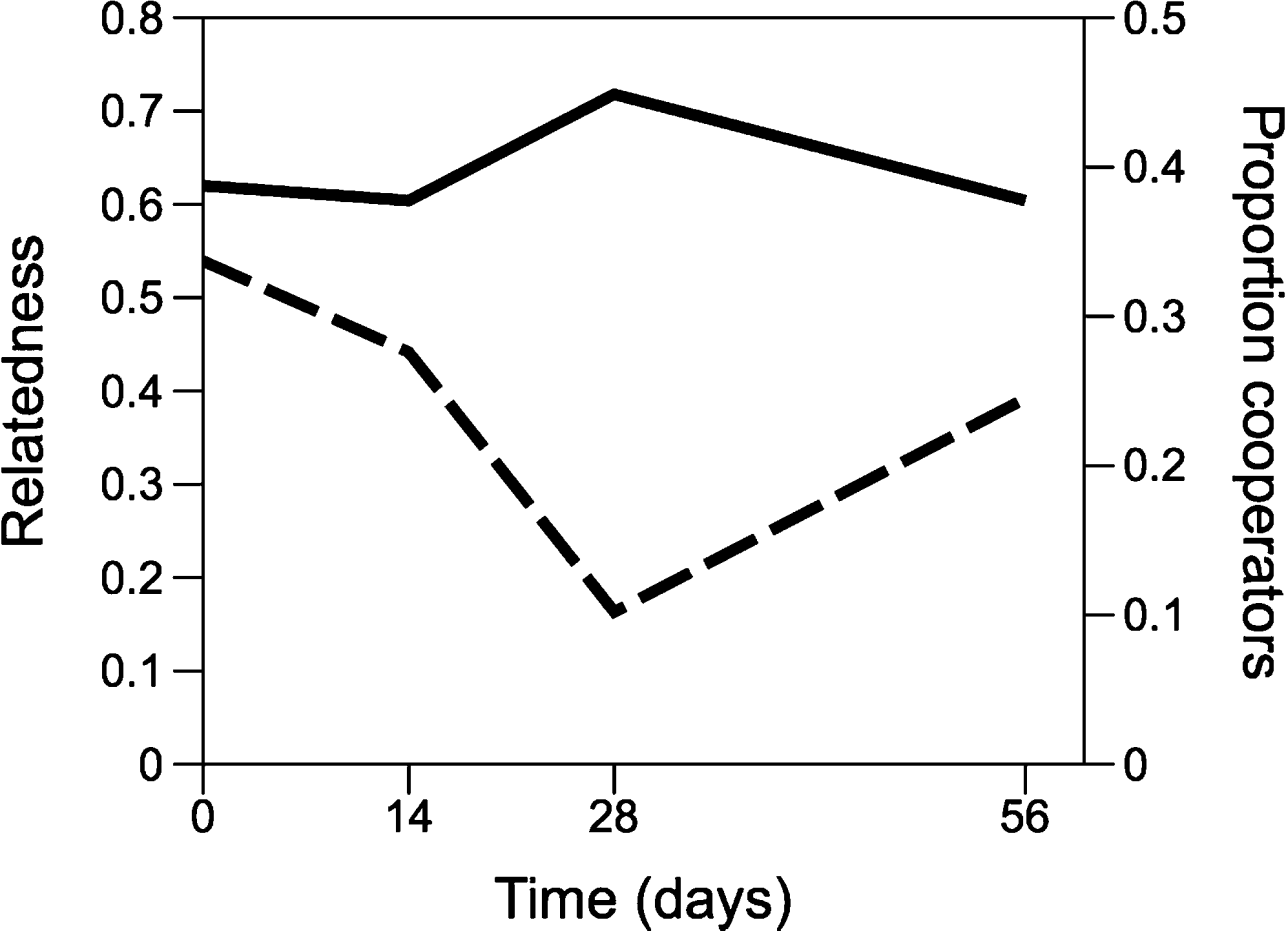
Population-level relatedness (solid line) remained high over the course of evolution in Raymond et al.'s semi-natural experimental plot. High relatedness is predicted by theory to maintain cooperation; Cry producers (dashed line) did indeed persist in this experiment. (Redrawn from Fig. 4B in 39 with permission from AAAS.)

In their study, Raymond et al. 39 showed that Cry cooperators and cheats both persisted in the population over 56 days of natural transmission and evolution, albeit with cooperators in the minority (Fig. 1). Coexistence was partially due to a negative correlation between relative fitness and relative frequency for both genotypes, but Raymond et al., showed that this was reinforced by a further level of self-regulation: the presence of cheats reduces overall population growth, but cooperators gain an extra fitness advantage at low density and this helps them ‘bounce back’ from cheat invasion. (It should, however, be noted that these relationships break down when only very small numbers of cooperators are present, as a threshold amount of Cry is required to rupture the host gut and allow access to the haemocoel.) Further, over the 56 days, population-level relatedness remained high (Fig. 1) and the distribution of bacteria on leaves was highly aggregated. Thus, two demographic conditions necessary for cooperation – high relatedness and patch structure – were maintained in this semi-natural system.

## How do other selection pressures acting within hosts and/or reservoirs affect selection on cooperation?

Finally, we must recognise that selection on other traits can mediate the evolution of a cooperative behaviour of interest. For instance, the invasion of a population of cooperators by a novel cheating mutant (or vice versa) could be curtailed under strong selection for non-social traits, simply because mutations favourable under the non-social selection pressure are more likely to arise in a cooperator cell, as their population size is so much larger. This was recently demonstrated by Morgan et al. 43, who added bacteriophage to mixed populations of wild-type *Pseudomonas fluorescens* and an isogenic mutant that does not produce the siderophore pyoverdine. Conditions were such that siderophores were beneficial (iron was limiting) and accessible to cheats (liquid growth medium allows free diffusion of siderophores), and cooperation was not under positive social selection (competition was entirely within-patch). The relative fitness advantage of cheating was reduced by the presence of phage over a range of initial cheat frequencies; from an initial frequency of 1%, cheats could successfully invade in the absence of phage but were outcompeted by cooperators when phage were added. In the presence of phage, clonal interference between strongly favoured phage resistance and weakly favoured social cheating placed rare cheats at a disadvantage, as they were less likely to also carry resistance mutations.

This is not the only way in which selection on non-social traits can affect the evolution of cooperation: my own work has shown that selection for hypermutability – a phenotype often observed in infections due to immune activity and prophylaxis 44 – can accelerate the breakdown of cooperation, as hypermutable clones are more likely to generate cheating mutants, have increased access to high-fitness cheating genotypes and reduce within-population relatedness 45, 46. Finally, pleiotropy can mean that cheating incurs a cost because it has deleterious effects on a second behaviour necessary for growth or persistence. One example of this is the poor ability of siderophore-deficient cheats of *P. aeruginosa* to form biofilms 47, 48; this could help to maintain siderophore cooperation when both siderophores and biofilm are beneficial.

## Conclusion: Taking results from the lab into the wild… and into the clinic?

Raymond et al.'s study showed that the social dynamics of Cry toxin production in a semi-natural field system recapitulated those observed in more strictly controlled laboratory conditions. More importantly, they demonstrated the continued coexistence of cooperators and cheats over many bacterial generations with natural transmission between hosts. However, to facilitate their experiment the authors excluded herbivores and natural predators of the insect host from their experimental plot; the effects of these on host population dynamics, transmission and bacterial population structure were therefore not investigated. Despite the exclusion of predators, the host population crashed towards the end of the experiment and more larvae were released; it is therefore unclear how well normal host population dynamics were captured. These caveats aside, researchers studying the links between pathogen cooperation and virulence should look to this study as an excellent example of what can be gained by studying microbial sociality outside the laboratory.

What light, then, do Raymond et al.'s results shed on the potential for exploiting bacterial cooperation for prophylactic benefit? In this particular case, both cooperators and cheats persisted in the population – unlike experiments which cleanly manipulate ecological variables in the lab (e.g. 7), neither genotype achieved fixation and cooperators gained an extra fitness advantage at low density. It is therefore unlikely that releasing large numbers of cheats into this system would cause a severe and terminal population crash, because cooperator clones have the capacity to recover from low density, cheat-dominated populations. This also has implications for the Trojan horse strategy, which relies on Trojan cheats sweeping to sufficiently high frequency that their removal represents a severe population crash: whether this could happen in Raymond et al.'s system is really not clear as cooperators were maintained at approx. 10–25% of the population. Finally, Raymond et al. did not address the extent and effects of coevolution between cooperators and cheats – although clones saved from their study could be used to explore this in future work. As in any antagonistic interaction, there exists the capacity for an ‘arms race’ to develop, whereby cooperators evolve to resist cheating and cheats evolve to better exploit cooperators (this has been shown empirically for biofilm formation in *P. fluorescens*: 49). If engineered cheats are to be used to destabilise infection populations, we must be sure that they retain the ability to coevolve with cooperators and do not fall behind in the arms race. Further, if social traits are encoded by mobile plasmids (as Cry toxins can be), then horizontal transmission can convert cheats to cooperators – the implications of this for the evolution of cooperation, and by extension for the Trojan horse strategy, have begun to be addressed by other authors 50–52.

More generally, it should be stressed that while much research has sought to explain how population structure affects selection for cooperation, remarkably little attention has focussed on how cooperation affects population structure. Three recent theoretical papers have suggested that selection for cooperation can concomitantly drive the evolution of population structures that support cooperation, reinforcing sociality via a positive feedback loop. Powers et al. 53 suggest that selection for cooperation can generate linkage disequilibrium between cooperative alleles and alleles that predispose the bearer to group living, while Van Dyken and Wade 54 explore how changes in levels of cooperation can affect the scale of resource competition. Further, Bonsall and Wright 55 show how cooperation can facilitate the evolution of resource specialisation, presumably limiting within-group competition. The exploration of possible bidirectional links between changes in population structure and relative benefits of cooperation would add enormous value to social evolution research in general, and research into the coevolution of microbial cooperation, virulence and epidemiology in particular. This has been alluded to in one guise in recent literature: the possible link between virulence factors as public goods and pathogen infective dose.

Pathogens that rely on virulence factors that act at a distance from the producing cell, and so are likely to function as public goods, tend to have a higher minimum infective dose than pathogens that use locally acting molecules (e.g. those injected directly into host cells) 56, 57; while the implications of this for virulence evolution are not clear cut, it is interesting that reliance on a potentially cooperative behaviour for virulence should place restrictions on the size of the bottleneck experienced at transmission. It is conceivable that placing a lower limit on the number of cells required to initiate infection could make clonal infection less likely and so make the presence of cheats in the population inevitable; however, it is equally conceivable that this could select for transmission via groups of related cells and so reinforce cooperation.

## Closing Remarks

Laboratory experiments have revealed the complexity of bacterial sociality, and the finding that various virulence factors are in fact cooperatively produced ‘public goods’ has spurred researchers on to suggest how we might exploit this for prophylactic gains. Given this, it is vital that we determine how well cooperation in the lab mimics the real situation in natural hosts and reservoirs. In order to do this, we must explicitly define and address a set of specific questions about the ecology and epidemiology of the pathogen under consideration. By exploring changes in cooperation and relatedness in a field experiment with a pathogenic bacterium, Raymond et al. help to frame these questions and set an example for future experimental work outside the confines of the laboratory.
